# Fatigue and failure mode analyses of glass infiltrated 5Y-PSZ bonded onto dentin analogues

**DOI:** 10.1038/s41598-024-64110-8

**Published:** 2024-06-10

**Authors:** Jonas Vinicius Meireles Rodrigues, Amir Mohidin Demachkia, Rita Adriana Souza da Silva de Assis, Mariana Marques Gomes, Tiago Moreira Bastos Campos, Kiara Serafini Dapieve, Luiz Felipe Valandro, Renata Marques de Melo

**Affiliations:** 1 Department of Dental Materials and Prosthodontics, Institute of Science and Technology, São Paulo State University (UNESP), Av. Engenheiro Francisco José Longo, 777, Jardim São Dimas, São José dos Campos, São Paulo 12245-000 Brazil; 2 Aeronautics Technological Institute (ITA), Praça Marechal Eduardo Gomes, 50, Vila das Acácias, São José dos Campos, São Paulo 12228-900 Brazil; 3 Post-Graduate Program in Oral Science, Faculty of Dentistry, Federal University of Santa Maria (UFSM), Av. Roraima, 1000, Cidade Universitária, Camobi, Santa Maria, Rio Grande do Sul 97105-900 Brazil

**Keywords:** 5Y-PSZ Zirconia, Experimental glass, Fatigue survival, Glass infiltration, Kaplan–Meier analysis, Structural reliability, Ceramics, Glasses, Mechanical properties

## Abstract

The purpose of this study was to evaluate the fatigue survival of 5Y-PSZ zirconia infiltrated with an experimental glass and bonded onto dentin analogues. Disc-shaped specimens of a 5Y-PSZ (Katana UTML Kuraray Noritake) were cemented onto dentin analogs (NEMA G10) and divided into four groups (n = 15): Zctrl Group (control, without infiltration); Zglz Group (Glaze, compression surface); Zinf-comp Group (Experimental Glass, compression surface); Zinf-tens Group (Experimental Glass, tension surface). Surface treatments were varied. Cyclic fatigue loading, oblique transillumination, stereomicroscope examination, and scanning electron microscopy were performed. Fatigue data were analyzed (failure load and number of cycles) using survival analysis (Kaplan–Meier and Log-Rank Mantel–Cox). There was no statistically significant difference in fatigue survival between the Zglz, Zctrl, and Zinf-comp groups. The Zinf-tens group presented a significantly higher failure load when compared to the other groups and exhibited a different failure mode. The experimental glass effectively infiltrated the zirconia, enhancing structural reliability, altering the failure mode, and improving load-bearing capacity over more cycles, particularly in the group where the glass was infiltrated into the tensile surface of the zirconia. Glass infiltration into 5Y-PSZ zirconia significantly enhanced structural reliability and the ability to withstand loads over an increased number of cycles. This approach has the potential to increase the durability of zirconia restorations, reducing the need for replacements and save time and resources, promoting efficiency in clinical practice.

## Introduction

Dental ceramics typically exhibit a flexural strength ranging from moderate to excellent. However they demonstrate low fracture toughness and may fracture when subjected to bending or when exposed to heat and cold^1^. To overcome this limitation, dental zirconia has emerged as a promising alternative. This material has superior mechanical properties, good chemical and dimensional stability, and an enhanced fracture toughness mechanism due to the tetragonal to monoclinic (t-m) phase transformation^2–4^.

Stawarczyk et al.^5^ categorized zirconia into distinct generations. The first generation exhibits a flexural strength exceeding 1000 MPa and is recommended for manufacturing fixed dental prosthetic frameworks in the posterior region On the other hand, the second-generation zirconia is partially stabilized with 3 mol% yttrium (3Y-TZP) and has smaller alumina grain sizes, resulting in improved optical properties and enhanced crystallographic stability. This generation is suitable for manufacturing monolithic restorations in the posterior region, although its optical characteristics are not ideal for replicating the natural translucency of the tooth in the anterior region^5,6^. Thus, the third generation of zirconia has emerged, stabilized with 5 mol% yttrium (5Y-PSZ) to maintain its crystals in the cubic phase, characterized by a larger grain size that allows for even light scattering and better transmission^7^.

The developments in zirconia mentioned above demonstrate efforts to create a material that combines high strength and translucency to meet market demands for esthetics and longevity^8^. However, it is unlikely for a single ceramic material to possess all these characteristics. Therefore, in recent years, many attempts have been performed to modify existing materials by incorporating new phases^9^. In this context, the process of glass infiltration into dental zirconia, proposed by Zhang and Ma^10^, stands out. This process involved applying a specific quantity of glass in paste form onto the surface of unsintered monolithic zirconia. This procedure has shown significant improvements in both adhesion and mechanical resistance of the material^10,11^.

Alongside this approach, Campos et al.^12^ conducted glass infiltration using the sol–gel method to enhance mechanical properties and adhesion to zirconia. Initially, the infiltration involved immersing unsintered zirconia in a silicic acid solution that gelled over time (5 days) and, upon drying, transformed into glass. Following zirconia sintering, the material reacted, forming zirconia silicate on the external surface of the sample while the remaining material retained its tetragonal zirconia state. In this scenario, material reliability increased (Weibull modulus ~ 15) without compromising mechanical strength (~ 900 MPa).

In a subsequent study by Ramos et al.^13^, the same process described by Campos et al.^12^ was replicated, with a reduction in infiltration time from 5 days to 40 min, and an analysis of interfacial fracture energy. The results indicated a higher interfacial energy for the infiltrated material compared to the material without glass infiltration^12–14^. Toyama et al.^15^ recommends conducting the glass infiltration on only one side of the restoration. This approach helps prevent distortions in the zirconia caused by possible faster cooling of the glass and disparities in thermal expansion/contraction coefficients^15^.

The literature has documented efforts to enhance the mechanical properties of zirconia, as well as uncertainties regarding which surface treatment would be most appropriate^8–13^. In this context, our study aimed to evaluate whether applying an experimental glass onto 5Y-PSZ could effectively enhance its mechanical properties, leading to increased fatigue resistance and improved structural homogeneity. Our null hypothesis (H0) was that the application of the experimental glass would not significantly affect fatigue survival compared to groups without the experimental glass.

## Materials and methods

This in vitro study assessed the fatigue resistance of 5Y-PSZ infiltrated with an in-house developed glass. The specimens were cemented onto dentin analogues, and the evaluation employed an accelerated cyclic fatigue approach. Detailed information about the materials, including manufacturers, composition, and batch numbers, can be found in Table 1.

**Table 1:** Information about the materials used in this study.

Commercial name	Manufacturer	Compositions	Batch
Katana UTML	Kuraray Noritake Dental Inc., Tokyo, Japan	ZrO_2_, Y_2_O_3_	125-3853
Cerabien ZR FC Paste Stain	Kuraray Noritake Dental Inc., Tokyo, Japan	Aluminum potassium silicate, glass, tin oxide, cerium (IV) oxide, glycerol, 1, 3-butanediol, and pigments	
NEMA G10	Trayout Usinagem Ferramentaria, Caçapava, Brazil	Continuous filament woven fiberglass sheet bonded with epoxy resin	
Multilink N	Ivoclar Vivadent, Schaan, Liechtenstein	Dimethacrylate, HEMA, Inorganic fillers, Barium glass, Ytterbium trifluoride, Spheroid mixed oxide	Y37993; Y16283; Y49510; Y06983
Clearfil ceramic primer plus	Kuraray Noritake Dental International, Okayama, Japan	3-Metacriloxipropil trimetoxissilano, 10-Metacriloiloxidecil Di-hidrogenofosfato (MDP), Etanol	320014
Hydrofluoric acid 40%	Developed by the author	40% Hydrofluoric acid (main component)	
Hydrofluoric acid 10%	Condac Porcelana, FGM, Joinville, SC, Brazil	10% Hydrofluoric acid (main component)	060821; 280721; 060821; 282721

### Zirconia discs fabrication

Partially yttrium-stabilized zirconia (5Y-PSZ) blocks (Katana UTML A1, Kuraray Noritake Dental Inc., Tokyo, Japan) were machined into cylindrical shapes following ISO 6872-2015 standards. Subsequently, these cylinders were transformed into discs (N = 60) using a precision cutting machine equipped with a diamond disc under water cooling conditions (Isomet 1000, Buehler, Lake Bluff, Illinois, USA). The resulting discs underwent polishing with #600 aluminum oxide sandpaper and #1200 silicon carbide sandpaper (Norton, Saint-Gobain Abrasivos, São Paulo, Brazil) using a water-cooled polisher (EcoMet, Buehler, Lake Bluff, Illinois, USA). The final dimensions were set at 1.5 × 15 mm, which is 20% larger than the ultimate target to account for sintering shrinkage.

Following these manufacturing steps, the discs underwent a cleaning process in a distilled water ultrasonic bath for 120 s (Cristófoli Ultrasonic Washer, Campo Mourão, Paraná, Brazil) and were subsequently dried.

**Figure 1:**
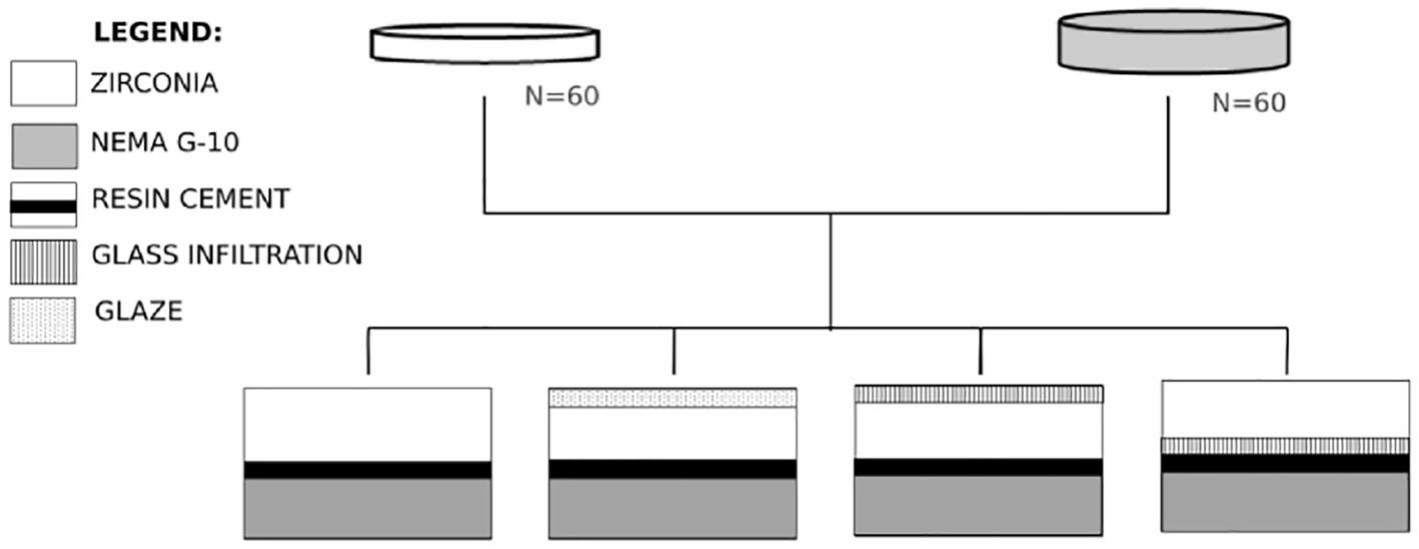
Experimental design of group division.

The specimens were then randomly divided into four groups (n = 15) based on the glass infiltration side, as illustrated in Fig. 1: Group Zctrl (control): 5Y-PSZ without glaze; Group Zglz: 5Y-PSZ with glaze on the occlusal surface; Group Zinf-comp: 5Y-PSZ with glass infiltration on the occlusal surface; Group Zinf-tens: 5Y-PSZ with glass infiltration on the cementation surface.

### Epoxy resin discs fabrication (NEMA G10)

Sixty NEMA G10 epoxy resin discs with dimensions of 2.5 mm in height and 12 mm in diameter were obtained and polished with #1200 silicon carbide sandpaper under water in a polishing machine (EcoMet, Buehler, Lake Bluff, Illinois, USA). These resin discs were then randomly paired with the ceramic discs.

### Production and infiltration glass application

The glass was synthesized by the sol–gel method. The source of silica used was silicic acid, obtained through the passage of a 10% aqueous solution of sodium silicate (at 100 °C for 24 h), following the methodology developed by Campos et al.^12,16^. The corresponding salts were added to form the glass with the following final composition: SiO_2_-68%, Al_2_O_3_-11.7%, CaO-3.0%, Na_2_O-7.3% and K_2_O-10.0%.

This composition was obtained by mixing the nitrates corresponding to the cations and the silicic acid solution corresponding to the silica. Then the material was calcined in a Vita Zyrcomat oven (Vita Zahnfabrik, Bad Sackingen, Germany) for 5 h at 650 °C. After calcination, the resulting material was ground and sieved (200 mesh). The glass was in a powder consistency and was mixed with Propylene glycol (Labsynth, São Paulo, Brazil), 1 g of glass and 0.5 mL of propylene glycol and homogenized with a non-metallic spatula for 60 s and applied to the sample with brush #1 (Tigre, Rio Claro, São Paulo, Brazil).

### Thermal treatment

The sintering process for Groups Zctrl and Zglz involved sintering according to the manufacturer's guidelines in a Sirona in Fire HTC furnace (Sirona Dental System GmbH, Bensheim, Germany) at 1550 °C for 2 h. In contrast, the glass-infiltrated zirconia Groups Zinf-comp and Zinf-tens underwent a sintering process developed by the authors. These samples were sintered at 1450 °C for 60 min, with a heating rate of 10 °C per minute and a cooling rate of 10 °C per minute.

After sintering, all samples were polished for 120 s using #1200 silicon carbide sandpaper (Norton, Saint-Gobain Abrasivos, São Paulo, Brazil) under water, employing a polishing machine (EcoMet, Buehler, Lake Bluff, Illinois, USA). Subsequently, they underwent a 480-s cleaning process in an ultrasonic bath filled with distilled water (Ultrasonic Washer Cristófoli, Campo Mourão, Parana, Brazil) and were dried using gauze. The glass was applied to the tensile or compression ceramic surface using a #1 brush (Brush 815, Tigre, Rio Claro, São Paulo, Brazil) until the entire surface was thoroughly and uniformly coated. Following this, they were placed in the sintering furnace and sintered at 1550 °C for 120 min, with a heating rate of 10 °C per minute and a cooling rate of 10 °C per minute.

### Cementation procedure

The cementation surface of zirconia (5Y-PSZ) specimens in groups “Zctrl”, “Zinf-comp”, and “Zglz” were sandblasted with 50 µm aluminum oxide particles (BioArt, São Paulo, Brazil) for 10 s at a distance of 15 mm with 2 bars of pressure (Essence Dental, Araraquara, São Paulo, Brazil). The “Zglz” group received a layer of Cerabien glaze (Kuraray Noritake Dental Inc., Tokyo, Japan) on its occlusal surface, followed by firing in the Vita Zyrcomat furnace (Vita Zahnfabrik, Bad Sackingen, Germany). The firing process involved two steps, first at 500 °C for 4 min, and then at 900 °C for 1 min, with a gradual temperature increase of 80 °C per minute over 3.15 min. The “Zinf-tens” group was conditioned with 40% hydrofluoric acid for 360 s and then washed with water for 30 s and dried with air jets. Subsequently, they cleaned in an ultrasonic bath of isopropanol for 480 s and air dried (Cristófoli, Campo Mourão, Paraná, Brazil). After that, Clearfil Ceramic Primer Plus (Kuraray Noritake Dental Inc., Tokyo, Japan), was vigorously applied with a microbrush on the cementation surface of all for 15 s and left to act for 60 s.

The cementation surface of NEMA-G10 epoxy resin samples (Trayout Usinagem Ferramentaria, Caçapava, Brazil) were etched with 10% hydrofluoric acid (Condac Porcelana, FGM, Joinville, Santa Catarina, Brazil) for 60 s. Afterward, they were rinsed with water for 30 s, air-dried, and then cleaned in an ultrasonic bath filled with isopropyl alcohol for 480 s (Cristófoli, Campo Mourão, Paraná, Brazil). Following this, Multilink Primer A + B adhesive/primer (Ivoclar Vivadent, Schaan, Liechtenstein) were mixed following the manufacturer’s instructions and vigorously applied on the epoxy resin cementation surface for 20 s, allowing it to act for 60 s.

After surface treatment, the resin cement Multilink N (Ivoclar Vivadent, Schaan, Liechtenstein) was evenly applied to both the treated ceramic surfaces and the corresponding epoxy discs. Subsequently, the ceramic discs were carefully positioned onto the epoxy discs under a constant load of 750 g, ensuring a standardized cement film thickness. Any excess cement was meticulously removed, and photoactivation was carried out using the Bluephase LED light-curing unit with an intensity of 1400 mW/cm^2^ (Ivoclar Vivadent, Schaan, Liechtenstein). The light application occurred in four different positions around each specimen, with each position receiving light for 40 s. Finally, the specimens were stored in distilled water at room temperature for 7 days (Table 2).

**Table 2:** Groups’ description and their respective surface treatments before cementation and Step-stress fatigue test.

Group	Infiltration	Infiltrated surface	Surface treatment
Zctrl	No	–	Sandblasting with aluminum oxide
Zglz	No	–	Sandblasting with aluminum oxide
Zinf-comp	Yes	Occlusal	Sandblasting with aluminum oxide
Zinf-tens	Yes	Intaglio	Hydrofluoric acid 40%

### Cyclic fatigue test (stepwise)

The cemented specimens underwent an cyclic fatigue test in a chamber filled with distilled water, utilizing an electrodynamics testing machine (Instron ElectroPuls E3000, Instron Corporation, Norwood, MA, United States) equipped with a 40 mm diameter stainless-steel hemispherical piston, operating at a frequency of 20 Hz^16,17^. The load was centrally applied along the long axis of the specimen. To ensure optimal stress distribution and prevent surface contact damage, a layer of non-rigid cellophane paper (2.5 µm) was interposed, and a thin adhesive tape (110 µm) was positioned between the piston and the ceramic surface^18^.

The cyclic fatigue test adhered to specific parameters: an initial load of 200 N, a consistent increment of 200 N, and duration of 10,000 cycles. Initially, a 200 N load was sustained for 5000 cycles to allow the piston to settle on the specimen. Subsequently, the load increased incrementally by 200 N every 10,000 cycles, reaching loads of 400 N, 600 N, and so forth, up to 2800 N, until the specimen fractured, or until 135,000 cycles were achieved^19^. At the conclusion of each step, oblique transillumination was employed to visually identify any failure. If no failure was observed, the test continued. Data on fatigue failure load (FFL) and the number of cycles for failure (CFF) at the moment of failure were documented for statistical analysis.

### Fractographic

The specimens that experienced failure were sectioned perpendicular to the crack under water, employing a diamond cutting disc (Isomet 1000, Buehler, Lake Bluff, Illinois, USA). Fractographic marks and the fracture origin were subsequently identified under a stereomicroscope (Stereo Discovery V20, Zeiss; Niedersachsen, Gottingen, Germany)^20^. For more in-depth analysis, two representative specimens from each group were chosen and examined using a scanning electron microscope (SEM) (Inspect50S, FEI; Brno, Czech Republic).

### Statistical analysis

Statistical software (IBM SPSS Software; IBM, Armonk, USA) was used for statistical analysis. Fatigue data (fatigue failure load and number of cycles for failure) were analyzed by Kaplan–Meier post-hoc tests and Mantel–Cox (Log-Rank) to assess the mean, confidence interval, and survival probability throughout the testing phases^21^.

To test the null hypothesis that there would be no significant difference in fatigue survival between the groups, the Log-Rank Mantel–Cox test was used. This statistical method used allows for a comprehensive assessment of survival data, particularly in scenarios where comparison of survival curves is essential. Through this analysis, we aimed to determine whether experimental glass infiltration into zirconia effectively increases its structural reliability and load capacity under cyclic fatigue conditions.

### Ethical approval

This article does not contain any studies with human participants performed by any of the authors.

### Informed consent

For this type of study, formal consent is not required.

## Results

### Cyclic fatigue (stepwise)

Cyclic fatigue results revealed lower values for fatigue failure load (FFL) and number of cycles to failure (CFF) in the control group (Zctrl). Intermediate values were observed for 5Y-PSZ with glaze (Zglz) and for 5Y-PSZ infiltrated with glass on the compression surface (Zinf-comp). The highest values were found in 5Y-PSZ infiltrated with glass on the tensile surface (Zinf-tens). Pairwise comparisons indicated that glass infiltration on the tensile surface of 5Y-PSZ (Zinf-tens) significantly increased both FFL and CFF when compared with 5Y-PSZ infiltrated with glass on the compression surface (Zinf-comp), 5Y-PSZ with glaze (Zglz) and the control group (Zctrl) (see Table 3).

**Table 3:** Mean fatigue failure loads (FFLs) in Newton and the number of cycles to failure (CFF), with respective 95% confidence interval (CI).

Group	FFL Mean (CI)	CFF Mean (CI)
Zctrl	933 (819–1048)^b^	78.3 (66.9–89.7)^b^
Zglz	1053 (993–1113)^b^	90.3 (84.3–96.3)^b^
Zinf-comp	1080 (950–1210)^b^	93.0 (79.9–106.0)^b^
Zinf-tens	1380 (1240–1520)^a^	123.0 (109.0–136.9)^a^

Different letters in each column indicate statistical differences as determined by Kaplan–Meier post-hoc tests and Mantel–Cox (Log-Rank).

To test the null hypothesis, the Log-Rank Mantel-Cox test was used. The results of the pairwise comparisons between groups are summarized below:

For the Log Rank test, the p-value for Zglz was 0.178, indicating that there was no significant difference between Zctrl and Zglz. However, for Zinf-tens, the p-value was 0.0001, indicating a significant difference between Zctrl and Zinf-tens. When comparing Zglz and Zinf-tens relative to Zinf-comp, the p-values were 0.235 and 0.0001, respectively. This shows no significant difference between Zinf-comp and Zglz, but a significant difference between Zinf-comp and Zinf-tens.

Furthermore, the p-value between Zglz and Zinf-tens was 0.009, indicating a significant difference in the survival curves between these groups. As the significance level is less than 0.05, it suggests that there is strong evidence to reject the null hypothesis, signifying that the survival probabilities between these groups are different.

In conclusion, Zinf-tens differs significantly from both Zctrl and Zinf-comp. However, there are no significant differences among Zctrl, Zinf-comp, and Zglz. Notably, there is a significant difference between Zglz and Zinf-tens.

**Figure 2:**
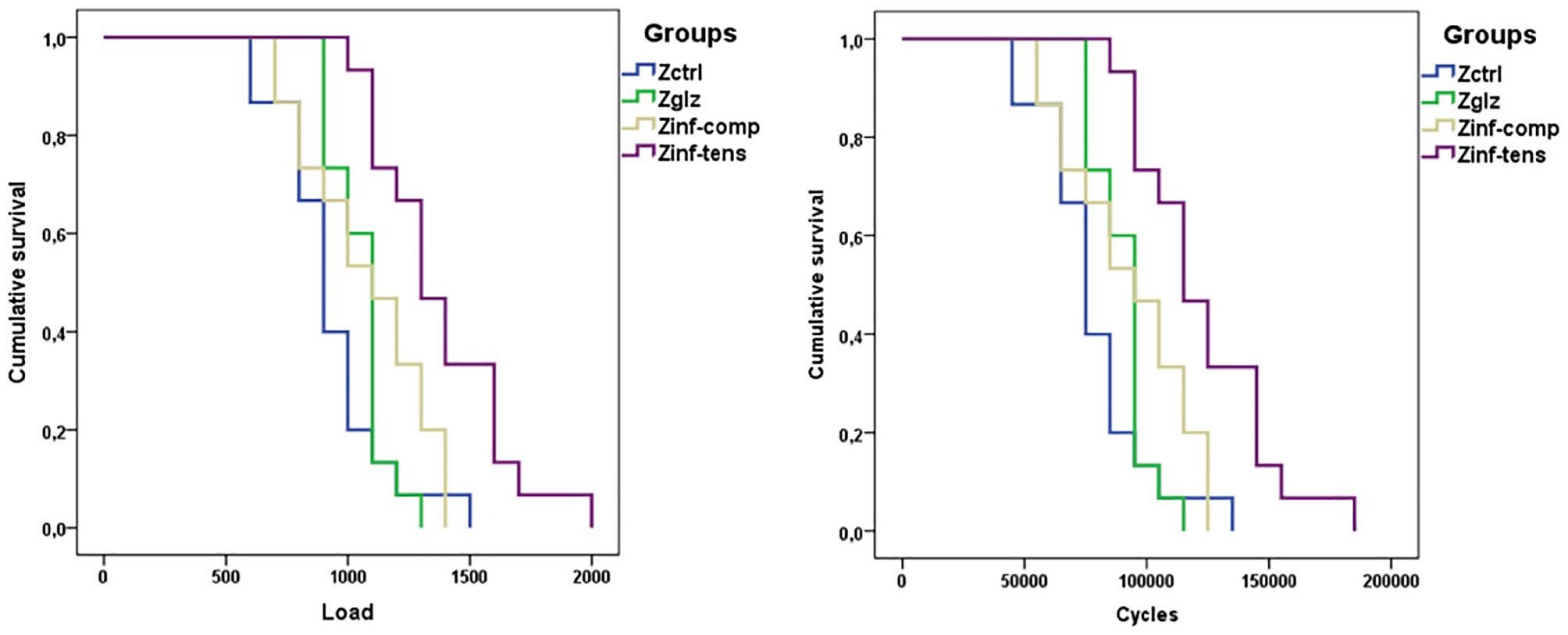
Kaplan–Meier curves for the fatigue survival analysis based on applied load and number of cycles to failure.

The survival analysis indicated that glass infiltration on the tensile (cementation) surface led to a higher survival rate compared to infiltration on the compression (occlusal) surface. Furthermore, the groups with glaze and without glass infiltration showed a lower survival rate when compared to the group with glass infiltration on the tensile surface (refer to Fig. 2).

### Fractographic analysis

**Figure 3:**
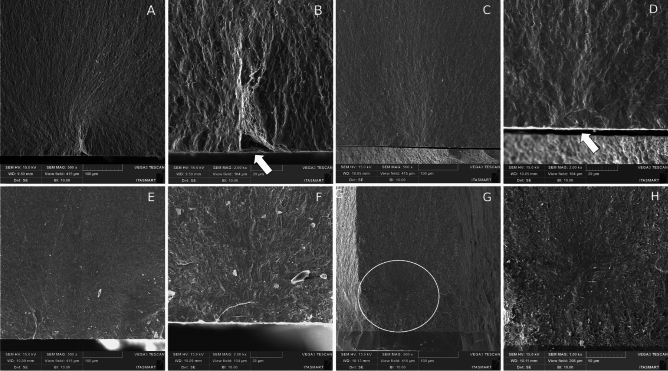
The micrographs of the failure modes for Zctrl (**A**, **B**), Zglz (**C**, **D**), Zinf-comp (**E**, **F**), and Zinf-tens (**G**, **H**).

Figure 3A and B show the micrographs of the failure modes for the Zctrl group. Figure 3A displays the fracture mirror origin followed by hackle lines. In Fig. 3B, one can clearly observe the fracture origin (white arrow) that occurred on the tensile surface, followed by hackle lines. For group Zglz (Fig. 3C,D), where glaze was applied to enhance the restoration’s appearance on the external surface, fracture origin can be observed at the tensile surface followed by heckle lines indicating the direction of crack propagation. In Fig. 3D, it can be noticed that the fracture origin (white arrow) is more discreet compared to the Zctrl group. Figure 3E and F represent the Zinf-comp group, which received glass infiltration on the compression surface (occlusal). The fracture patterns for this group were similar to those Zglz group.

Regarding the Zinf-tens group, glass was infiltrated into the tensile surface (intaglio surface) to create a graded modulus of elasticity. The failure mode in this group exhibited a distinct pattern compared to the other groups and differed from that of ceramic materials. The fracture origin did not occur on the tensile surface but rather within the material itself. The crack was unable to propagate through the infiltrated glass and the layers it formed, resulting in the fracture initiating from internal defects inherent to the material, such as pores and flaws commonly encountered during material processing. In (Fig. 3G), the fracture origin (white circle) can be observed within the material, along with the layer created by the glass. The fracture origin in the tensile region is not visible. In Fig. 3H, it displays the same structures mentioned earlier, including the hackle pattern formed during bending and the flaw/porosity that initiated the failure.

## Discussion

We anticipated a null hypothesis (H0) assuming that the application of the experimental glass onto 5Y-PSZ would not have a significant effect on fatigue survival compared to groups that did not receive the experimental glass treatment. However, our study findings provided evidence to reject this null hypothesis. Specifically, the group in which the experimental glass was infiltrated into the tensile surface of the zirconia (Zinf-tens) exhibited a significantly higher failure load and a distinct failure mode compared to other groups. This indicates that the experimental glass did indeed have a significant impact on fatigue survival, enhancing the structural reliability of the zirconia and altering its failure mode.

### Glass infiltration

**Figure 4:**
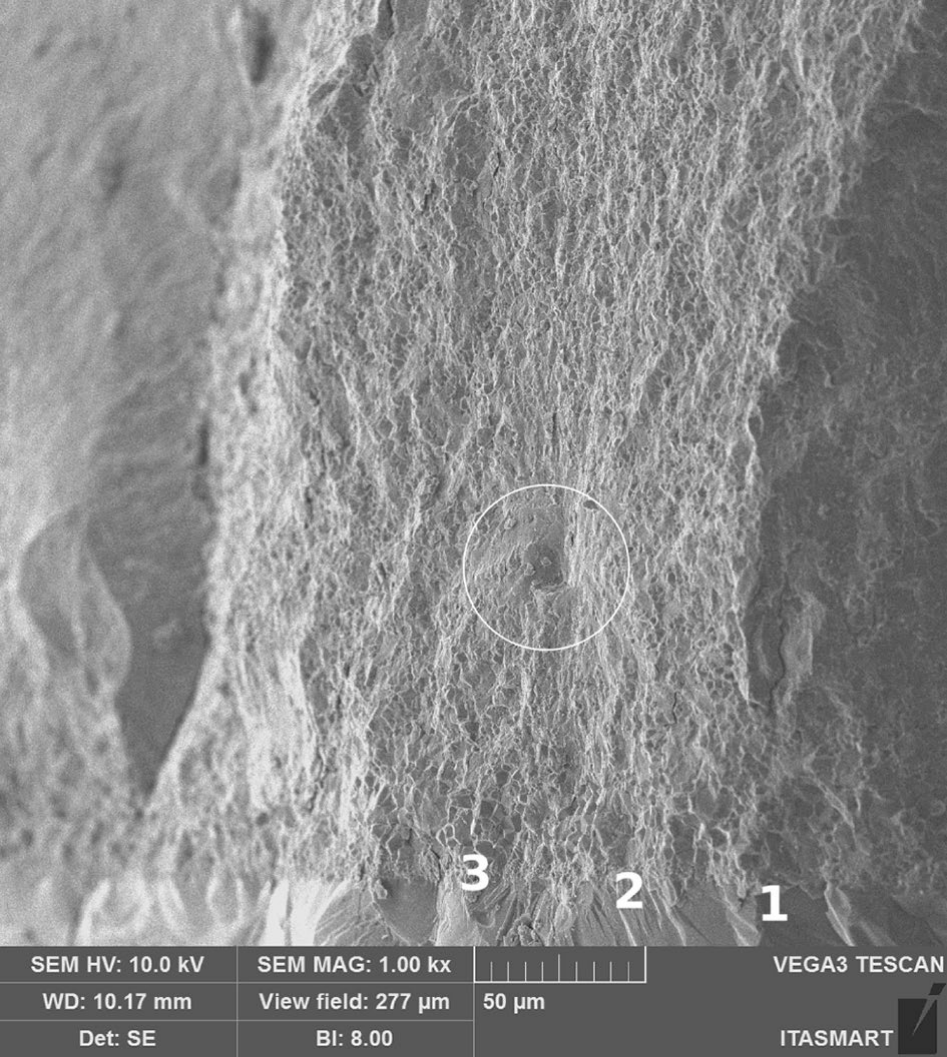
Scanning electron microscopy (SEM) images of the Zinf-tens group. Micrograph of the defective origin of the fracture (white circle), 1-infiltrated glass, 2-smaller cubic grains caused by glass infiltration, 3-cubic zirconia, highlighting the distinct failure mode pattern compared to the other groups, with the failure origin within the material, magnification 1000×.

All groups tested in this study share the same chemical composition characteristics: 70.6 wt.% cubic grains, 28.9 wt.% tetragonal grains, and 0.2 wt.% monoclinic grains^22,23^. Notably, the cubic grains in this zirconia stand out due to their relatively large dimensions, measuring 5 µm. In Fig. 4, the fracture origin (white circle) is visible within the material, along with the layer created by the glass infiltration in the Zinf-tens group (where glass was infiltrated into the tensile surface).

In a study conducted by Ramos et al.^13^, the authors infiltrated second-generation zirconia (3Y-TZP) with glass. While the infiltrated zirconia showed higher interfacial energy compared to the non-infiltrated one, the infiltration did not occur uniformly, resulting in gaps and pores where the material's toughening mechanism performed. This made it susceptible to Low-Temperature Degradation (LTD). This outcome contrasts with our results, and it could be attributed to the high content of yttrium stabilizing oxide. A higher stabilizer content results in a lower transformation rate^24^.

This is one of the reasons why third-generation zirconia exhibits lower strength, given its limited toughening ability. Reis et al.^25^ employed a similar infiltration technique to that of Ramos et al.^13^ and observed improved structural homogeneity and hardness, along with the formation of zirconium silicate on the surface. Campos et al.^26^ successfully reduced the transformation rate (t-m) after aging 3Y-TZP zirconia through enhancements in the infiltration technique, thus increasing the material's longevity. Rodrigues et al.^22^ demonstrated that the infiltration method involved applying a glaze-like paste, allowing for uniform infiltration in the desired region, resulting in an smooth and uniform surface, potentially ideal for preventing excessive wear on opposing teeth or microbial adhesion^22^.

Furthermore, in our study we observed the formation of zirconium silicate (ZrSiO4) in the infiltrated region. Clinically, this could play a crucial role in reducing low-temperature degradation (LTD) due to the material's inherently low t-m transformation rate, potentially extending restorations’ longevity.

From a clinical standpoint, it is also important to consider the thickness of the glass layer. In a previous study conducted by our research group, we utilized the same infiltration glass and conducted a microstructure evaluation. In this study, the thickness of the glass was circa 25–30 µm (measured at SEM images)^22^. This seems minimal compared to the internal relief of prosthetic crowns, but we are currently conducting another in vitro study in which the internal adaptation of glass-infiltrated zirconia restorations in extracted teeth, as well as the fatigue behavior and fracture mode are being assessed.

### Thermal treatment

In this study, the success of the infiltration can be attributed to a sintering protocol developed by our team, which differs from the manufacturer's recommended method. While some authors advocate for a vacuum furnace to eliminate gases and contaminants during sintering^27^, others suggest slow cooling to reduce residual thermal stress^28^. The developed method shows great promise, as thermal incompatibility leading to material failures is a common issue, especially with glaze^29^. Remarkably, this problem did not occur with the method devised by the authors.

### Cyclic fatigue test (stepwise)

In the current study, we developed an in-house infiltration glass for third-generation zirconia and assessed its performance using an accelerated life test on simplified disc models. The achieved results were promising, particularly in terms of the site of infiltration and material gradation. Thermal incompatibility issues associated with glaze on polycrystalline materials, as reported by some authors^30^, were successfully addressed in our study. We observed a flawless interaction between the experimental glass and zirconia, exemplified in Fig. 3G, illustrating how the glass seamlessly penetrated and bonded with the material. The infiltration was confined to one face of the material, effectively preventing distortions, a phenomenon also observed by Toyama et al.^15^.

Unlike glass ceramics, zirconia is not susceptible to etching by hydrofluoric acid, a common technique for creating surface roughness and improving adhesion to various substrates^31^. Instead, the manufacturer suggests aluminum oxide sandblasting (Kuraray Noritake, Japan),which is a controversial procedure. However, in second-generation zirconia, this sandblasting procedure activates the toughening mechanism, facilitating the t-m transformation and thereby enhancing its strength^32^.

Existing literature emphasizes the detrimental effects of this procedure on third-generation zirconia (ultra-translucent zirconia)^7,16^. This is attributed to the lower content of tetragonal grains in third-generation zirconia, resulting in a decreased t-m transformation^24^. In our study, we followed a conventional approach to glass infiltration, applying it externally as glaze and also on the cementation surface. This choice of method is in line with the findings of Della Bona et al.^1^ and Prado et al.^33^, who argue that the tensile side of the material significantly influences its fracture resistance.

The control group (Zctrl) exhibited a failure load of 933 N, followed by Zinf-comp with 1053 N and Zglz with 1080 N. Notably, both Zinf-comp and Zglz exhibited similar fractographic characteristics, with fractures originating from the tensile surface. Among these groups, Zglz, treated with the commercial Cerabien glaze from Kuraray Noritake, Japan, had a slightly higher failure load compared to Zinf-comp, which received the infiltrated glass.

The fractographic features observed in the 5Y-PSZ group that experienced glass infiltration in a prior study by Rodrigues et al.^22^ were not identified in the Zinf-comp group in the current investigation, despite both undergoing similar glass infiltration on the occlusal surface. In both studies, fractures originated from the tensile surface, but the distinctive crack line presented in the earlier study was absent in the present research.

This discrepancy could be attributed to the testing methodology. In the earlier study, specimens underwent a biaxial flexure test (ISO 6872:2015)^22,34,35^, where the applied load continued until catastrophic material failure. In contrast, in the present study, specimens were subjected to cyclic loading (stepwise) and assessed for any indications of failure at the conclusion of each cycle. Consequently, catastrophic material failure may not have occurred, and only a crack may have developed.

The Zinf-tens group, however, demonstrated a failure load of 1380 N, revealing statistically significant distinctions from the other groups. The purpose behind infiltrating glass on the tensile surface aimed to circumvent the adverse effects of sandblasting particles, as elucidated by Mao et al.^36^ and Inokoshi et al.^37^, and to achieve a gradual transition of the elastic modulus, akin to the Dentinoenamel Junction (DEJ). In Fig. 4, the layer arrangement is evident: glass (1), a layer of smaller cubic grains (2), and the actual cubic grains (3), accompanied by the resin cement just below the glass. This configuration resulted in the failure origin within the material, diverging from the failure mode observed in the other groups and the conventional failure mode in ceramic materials, where failure typically occurs on the tensile surface.

These results are highly promising as they expand the potential applications of ultra- translucent zirconia in dentistry, bringing them closer in strength to second-generation zirconia while obtaining superior aesthetic characteristics. Nevertheless, the surface treatment of the developed glass presents a significant challenge, as it requires a 40% hydrofluoric acid application, which carries a higher risk of manipulation and has limited accessibility, which should be investigated further^38,39^.

It can be stated that the infiltration of the developed glass, into ultra-translucent zirconia, improves the mechanical properties of zirconia, reduce defects caused by surface treatments and inherent material flaws, as well as increases the fatigue longevity.

## Conclusion

The conclusions drawn in the present study were:

1. The experimental glass successfully infiltrated third-generation zirconia, inducing a change in the failure mode (fracture origin shifted from the tensile surface to the interior, in the bulk of the material).
2. Glass infiltration on the tensile side of the discs led to the formation of a graded zirconia, contributing to the improvement of long-term fatigue resistance in the infiltrated groups.

Our results highlight the positive impact of the experimental glass on zirconia properties, suggesting potential advancements in structural applications. This approach is recommended to achieve better fatigue performance and prolonged material longevity resulting from the increased resistance caused by the experimental glass.

## Data Availability

The experimental data and results supporting the conclusions of this study are available in the online data repository of São Paulo State University “Júlio de Mesquita Filho” (UNESP): https://repositorio.unesp.br/items/39817f65-dce9-499e-b406-70eb6d5755dc/full. They are also available upon request to the corresponding author.
